# Body Mass Index and Risks of Incident Ischemic Stroke Subtypes: The Japan Public Health Center-Based Prospective (JPHC) Study

**DOI:** 10.2188/jea.JE20170298

**Published:** 2019-09-05

**Authors:** Yuanying Li, Hiroshi Yatsuya, Hiroyasu Iso, Kazumasa Yamagishi, Isao Saito, Yoshihiro Kokubo, Norie Sawada, Shoichiro Tsugane

**Affiliations:** 1 Department of Public Health, Fujita Health University, Aichi, Japan; 2 Department of Public Health and Health Systems, Nagoya University Graduate School of Medicine, Nagoya, Japan; 3 Public Health, Department of Social Medicine, Osaka University Graduate School of Medicine, Osaka, Japan; 4 Department of Public Health Medicine, Faculty of Medicine, University of Tsukuba, Ibaraki, Japan; 5 Program for Nursing and Health Sciences, Ehime University Graduate School of Medicine, Ehime, Japan; 6 Department of Preventive Cardiology, National Cerebral and Cardiovascular Center, Osaka, Japan; 7 Epidemiology and Prevention Division, Research Center for Cancer Prevention and Screening, National Cancer Center, Tokyo, Japan

**Keywords:** body mass index, ischemic stroke subtypes, general population, cohort study, sub-distribution hazard ratio

## Abstract

**Background:**

The association of body mass index (BMI) with risks of ischemic stroke subtypes have not been established.

**Methods:**

Cumulative average BMI was calculated using self-reported body weight and height obtained from baseline (Cohort I in 1990, and Cohort II from 1993–1994) and 5- and 10-year questionnaire surveys of Japan Public Health Center-based prospective (JPHC) study. A total of 42,343 men and 46,413 women aged 40–69 years were followed-up for the incidence of lacunar, large-artery occlusive, and cardioembolic strokes. A sub-distribution hazard model was used to estimate sub-distribution hazard ratios (SHRs) and the 95% confidence intervals (CIs).

**Results:**

During a median of 20.0 years of follow-up, we documented 809 and 481 lacunar, 395 and 218 large-artery occlusive, and 568 and 298 cardioembolic strokes in men and women, respectively. After adjustment for baseline age, updated smoking, alcohol consumption, leisure-time physical activity, and histories of hypertension, dyslipidemia, and diabetes mellitus, cumulative average BMI was positively linearly associated with lacunar (trend *P* = 0.007), large-artery occlusive (trend *P* = 0.002), and cardioembolic (trend *P* < 0.001) strokes in men, and with lacunar (trend *P* < 0.001) and large-artery occlusive (trend *P* = 0.003) strokes in women. There were approximately two-fold excess risk of cardioembolic stroke in both sexes and of lacunar and large-artery occlusive strokes in women for cumulative average BMI ≥30 kg/m^2^ compared to BMI 23–<25 kg/m^2^.

**Conclusion:**

Cumulative average BMI showed a positive linear effect on sub-distribution hazards of lacunar, large-artery occlusive, and cardioembolic strokes in both sexes, except for cardioembolic stroke in women.

## INTRODUCTION

Ischemic stroke is one of the leading causes of long-term disability and mortality worldwide.^1^ Increased risk of ischemic stroke by excessive adiposity has been observed in diverse populations,^2^^–^^4^ but its associations with ischemic stroke subtypes (eg, lacunar, large-artery occlusive, and cardioembolic strokes) were examined only in a few studies^5^^,^^6^ and have not been established. One study in American communities (*n* = 13,549, baseline year: 1987)^5^ reported linear and positive associations of BMI with all ischemic stroke subtypes, which was totally explained by known risk factors (eg, blood pressure, diabetes, and high density lipoprotein cholesterol). Another study in Japan (*n* = 1,621, baseline year: 1961) did not find any associations except for a positive association between BMI and lacunar stroke in women, which was independent of known risk factors, including systolic blood pressure.^6^ One explanation for the discrepancies might be that the mean BMIs and the compositions of ischemic stroke subtypes differ considerably between the two populations.^7^^–^^9^ However, it might also be due to differences in the way BMI was modeled: baseline only in the former^5^ versus updated as time-dependent covariate in the latter.^6^ BMI used in the latter study would mainly reflect a short-term effect of the exposure since BMI obtained in the nearest examinations before the events of cerebral infarction was related to the outcome. This might be appropriate for an exposure like blood pressure, which would elevate generally with advancing age, but might not be for an exposure like BMI, which would change in different directions with aging. Instead, we defined cumulative index of BMI as a kind of time-dependent exposure in the present study since potential effect of BMI on the outcome would require longer time. Nevertheless, we carried out baseline BMI only or updated BMI analyses to facilitate comparisons among studies. Since cumulative average BMI was regarded to capture the average level of exposure over longer-term than a single measurement, such as baseline (long latent period) or update (short latent period), yielding useful information for prevention, the analysis using cumulative average BMI was considered as the main one.

Furthermore, we could not readily assume that similar observation would be made even if another study was conducted in Japan employing similar methodological approaches, since the mean BMI increased from 21.7 to 23.3 from 1960 to 1990 in men aged 50–59 in Japan.^10^^,^^11^ It would be more relevant to examine the association in a more contemporary cohort. In the present study, we examined the associations of BMI with ischemic stroke subtypes in a Japanese representative and more recent cohort. Since hypertension is the most significant risk factor for ischemic stroke, the stratified analysis by hypertension was also conducted to examine the possible effect modification.

## METHODS

### Population

The Japan Public Health Center-based prospective (JPHC) study was established in 1990 (Cohort I) and 1993–1994 (Cohort II) based on resident registry of 29 districts under 11 public health-center areas throughout Japan.^12^^,^^13^ Of 140,420 individuals enrolled (Cohort I, *n* = 61,595; Cohort II, *n* = 78,825), two public health-center areas without data on cardiovascular disease incidence (*n* = 23,524), individuals not in the target age range (Cohort I: 40–59 years, Cohort II: 40–69 years), non-Japanese, and those who moved out before baseline survey (*n* = 552) were excluded from the present analysis. A self-administered questionnaire regarding height, body weight, lifestyles, and medical histories was distributed to all participants at baseline and returned from 43,130 subjects in Cohort I and 51,948 in Cohort II. Subjects with missing information on height or body weight (*n* = 1,067); smoking, alcohol consumption, leisure-time physical activity, histories of hypertension, dyslipidemia, or diabetes mellitus (*n* = 1,753); as well as those who reported histories of cardiovascular disease or cancer (*n* = 3,502) at baseline were further excluded, leaving 42,343 men and 46,413 women (Cohort I: 40,563; Cohort II: 48,193) for the analysis. The study protocol of the JPHC study, was approved by the Human Ethics Review Committees of the National Cancer Center (13-021), Osaka University (14285-4), and Fujita Health University (HM15-255).

### Measurements of covariates

Follow-up questionnaire surveys were conducted at 5 and 10 years to update height, body weight, lifestyles, and medical histories. BMI was calculated as body weight (kg) divided by squared height (m^2^). The validity of self-report was examined in the JPHC Study participants (Cohort I) for whom health check-up data were available.^14^ Namely, the self-reported BMIs (mean: 23.45 in men and 23.57 in women) were slightly lower than measured BMIs (mean: 23.54 in men and 23.78 in women), and the Spearman correlation coefficients were 0.89 in men and 0.91 in women in the self-reported BMI range from 14 to 40 kg/m^2^. Smoking was categorized into never-smoker, ex-smoker, current smoker of <20 cigarettes/day, and current smoker of ≥20 cigarettes/day. Alcohol consumption habit was evaluated as the weekly consumption by multiplying weekly frequency and the amount of five specific alcoholic beverages taken on each occasion, which was grouped into never-drinker, former-drinker, and current drinker of <150 g/week, current drinker of 150–<300 g/week, and current drinker of ≥300 g/week. The frequency of leisure-time physical activity was assessed as none, 1–3 times/month, 1–2 times/week, 3–4 times/week, and almost everyday. Histories of hypertension, dyslipidemia, or diabetes mellitus were defined using the self-report of previous diagnoses or current use of medications, as well as by physical and laboratory data for those with blood pressure, total cholesterol, and casual glucose measurements at baseline (*n* = 32,292, 32,289, and 21,871 subjects, respectively) using the following criteria: systolic blood pressure ≥140 mm Hg or diastolic blood pressure ≥90 mm Hg; total cholesterol ≥240 mg/dL; and blood glucose ≥126 mg/dL for those fasting more than 8 hours (otherwise ≥200 mg/dL).

### Ascertainment of ischemic stroke event

All the major hospitals capable of managing acute stroke care located in the sampling areas of the study were registered. The medical records were reviewed regularly by physicians in registered hospital or public health center blinded to the risk factor statuses, using the standard format of registry. To complete surveillance for incidence of fatal stroke, a systematic review of death certificate was conducted. The stroke diagnosis and the incidence date was assigned according to criteria adopted from the National Survey of Stroke criteria, which require a constellation of neurological deficits of sudden or rapid onset lasting at least 24 hours or until death.^15^^,^^16^ A stroke was classified as ischemic stroke when acute infarction appeared on the computed tomography scans or magnetic resonance images, or documented in the autopsy report, and no evidence for intracerebral or subarachnoid hemorrhage. Ischemic stroke was further classified into lacunar, large-artery occlusive, and cardioembolic stroke. A stroke was classified as lacunar according to the location (basal ganglia, brain stem, thalamus, internal capsule, or cerebral white matter) of the infarct. Definition of large-artery occlusive stroke was defined as infarct involving cortical areas. Cardioembolic stroke depended on clinical diagnosis with the presence of an embolus in the brain or medical record evidence of a possible source of embolus such as moderate or more severe valvular heart disease, atrial fibrillation, or intracardiac thrombus. Undetermined ischemic stroke included all ischemic strokes that could not be classified into lacunar, large-artery occlusive, or cardioembolic stroke (*n* = 82 in men and *n* = 127 in women). They were censored at the time of onset and were not analyzed in relation to BMI in the present study. However, we conducted sensitivity analyses that regarded these undetermined events as lacunar, large-artery occlusive, or cardioembolic stroke. A focal neurological deficit lasting <24 hours (ie, transient ischemic attack) and asymptomatic lacunar stroke were not systemically ascertained and, thus, were not included. The reliability of the same coding system was evaluated in a different study.^16^

### Follow-up

Subjects were followed-up from the date of the baseline survey (cohort I in 1990, and cohort II 1993 to 1994) until first incidence of stroke regardless of the subtypes, death, moving out of study area, or the end of 2012, whichever came first. Residence and survival were ascertained annually using residential registries maintained by each municipality. In Japan, residency and death registration are required by law, and these registries are believed to be complete. Participants who moved out from their original residential areas were identified in each area and treated as censored at that time. Information on the cause of death was obtained through the death certificate provided by the Ministry of Health, Labor, and Welfare after the Ministry of Internal Affairs and Communications granted permission.

### Statistical analysis

Men and women were analyzed separately. The exposure variable (BMI) was treated in the analyses in three different ways. First, baseline BMI and covariates were used. Second, BMI was updated at 5- and 10-year follow-up. The covariates were also updated as time-dependent variables in the statistical models. Third, as the main analysis, cumulative average BMI was calculated for each subject using values in each 5-year interval questionnaire survey. Namely, it was defined as the values at baseline for those followed up for less than 5 years, as the mean of baseline and 5-year survey for those followed-up for 5 years or more but less than 10 years, and as the mean of baseline, 5-, and 10-year surveys for those followed for more than 10 years. Cumulative averages of numerical covariates were created in the same fashion. Categorical variables were simply updated to newer values. For those who were followed up for 5 years or more or 10 years or more but without 5-year or 10-year questionnaire data on those variables, the missing values were imputed using the last-observation carried forward method.

For all the BMI variables, they were divided into seven categories, <19, 19–<21, 21–<23, 23–<25, 25–<27, 27–<30, ≥30 kg/m^2^, with 23–<25 kg/m^2^ as the reference (a category which contains the mean BMI for men [23.5 kg/m^2^] and women [23.6 kg/m^2^]).

Since subjects who died would have no probability of experiencing incidence in the future, treating them as non-informative censoring would inflate the estimate of cumulative incidence. The magnitude of inflation in the incidence would be greater in categories with higher mortality rates (ie, in low and high BMI categories than in the reference one), resulting in overestimation of relative risks. Thus, deaths from causes other than ischemic stroke, as well as those from undetermined subtype of ischemic stroke, were considered as competing events in the present study. Fine and Gray’s sub-distribution hazard model was used to estimate sub-distribution hazard ratios (SHRs) and 95% confidence intervals (CIs).^17^^,^^18^ Three statistical models were performed stratified by 9 public health centers. Multivariable model 1 adjusted for baseline age (5-year categories from 40–44 to 65–69 years). Multivariable model 2 adjusted further for smoking, alcohol consumption, and leisure-time physical activity. Multivariable model 3 adjusted further for histories of hypertension, dyslipidemia, and diabetes mellitus. The SHR from Fine and Gray’s model denotes the magnitude of the relative change in the sub-distribution hazard function associated with one unit change in the given covariate.^17^ Positive SHR denoted as X can be interpreted as evidence that one unit increase in a coefficient is associated with a (X − 1) * 100% increase in the rate of an event of the interest in subjects who are either event-free or who have experienced a competing event. We described SHRs greater and smaller than unity (1) as increased and decreased SHR, respectively, in this study.

The stratified analysis by the history of hypertension, either at baseline, 5 years, or 10 years was done in multivariable model 3. Interaction of hypertension with BMI categories was tested.

For the updated BMI analyses, a time-dependent Cox proportional hazard model^19^ stratified by 9 public health centers was used to estimate hazard ratios (HRs) and 95% CIs for incidence occurred between each 5-year questionnaire cycle, after adjusting for time-dependent covariates.

Test of linear trend was performed by assigning median BMI values for all subjects in each BMI category and by treating it as a continuous variable. Test of quadratic trend was done by assigning squared values of median BMI.

All analyses were conducted using SAS for Windows, version 9.4 (SAS/STAT 13.1) (SAS Institute, Cary, NC, USA).

## RESULTS

Higher cumulative average BMI was associated with younger age in men but older age in women (Table 1). Current smoking was associated with lower cumulative average BMI in men. The prevalence of hypertension, hyperlipidemia, and diabetes were higher in men and women with higher categories of cumulative average BMI.

**Table 1.** Means and proportions of updated lifestyle and medical histories according to categories of cumulative average body mass index

	Body mass index, kg/m^2^							*P* for difference^a^

	<19	19–<21	21–<23	23–<25	25–<27	27–<30	≥30	
Men								
Number	1,712	5,753	11,007	11,989	7,342	3,720	820	
Age at baseline, years, mean (standard deviation)	54.1 (8.8)	52.7 (8.3)	52.1 (7.9)	51.4 (7.6)	50.8 (7.4)	50.5 (7.2)	50.1 (7.3)	<0.001
Age distribution, %								<0.001
40–44	19.3	21.9	22.0	23.2	25.4	25.8	29.9	
45–49	14.9	17.0	19.3	20.9	20.9	21.9	21.6	
50–54	15.1	17.8	19.0	20.6	21.1	21.5	18.7	
55–59	21.5	22.0	21.6	20.4	20.0	19.5	18.3	
60–64	12.0	10.7	9.7	8.5	7.8	7.2	8.3	
65–69	17.1	10.7	8.4	6.5	4.8	4.2	3.3	
Smoking, %								<0.001
Never-smoker	14.4	16.1	20.2	23.1	26.9	27.9	28.5	
Ex-smoker	29.0	30.9	33.4	37.1	37.1	36.5	34.2	
<20 cigarettes/day	22.3	17.7	14.5	11.0	9.6	7.4	7.3	
≥20 cigarettes/day	34.3	35.4	31.9	28.8	26.4	28.2	30.0	
Alcohol consumption, %								<0.001
Never-drinker	40.0	35.0	33.5	33.9	35.1	37.2	39.8	
Former-drinker	6.0	4.8	4.2	3.7	3.9	4.0	3.8	
<150 g/week	10.9	11.3	10.7	11.2	11.0	10.7	11.7	
150–<300 g/week	18.5	20.9	22.3	22.5	21.8	21.1	17.8	
≥300 g/week	24.7	28.0	29.2	28.6	28.2	27.0	27.0	
Leisure-time physical activity, %								<0.001
None	62.8	59.7	55.7	53.7	52.5	53.9	58.2	
1–3 times/month	14.0	16.3	18.9	20.0	20.6	21.1	19.4	
1–2 times/week	10.3	10.6	12.0	12.7	12.6	11.6	10.7	
3–4 times/week	6.3	6.5	6.6	6.9	7.8	7.3	6.2	
Almost everyday	6.7	7.0	6.7	6.7	6.6	6.1	5.5	
History of hypertension, %	22.0	25.5	29.5	34.7	39.6	45.3	49.9	<0.001
History of dyslipidemia, %	9.8	8.6	9.2	10.7	11.1	13.2	17.4	<0.001
History of diabetes mellitus, %	3.7	4.5	6.9	9.2	11.8	12.3	14.5	<0.001
Women								
Number	2,306	6,796	11,996	11,742	7,572	4,546	1,455	
Age at baseline, years, mean (standard deviation)	52.9 (8.7)	51.5 (8.3)	51.8 (8.0)	52.1 (7.7)	52.7 (7.7)	52.7 (7.6)	52.7 (7.6)	<0.001
Age distribution, %								<0.001
40–44	22.3	26.5	23.4	20.6	18.4	18.1	18.4	
45–49	17.1	19.4	20.1	19.9	18.2	17.7	18.7	
50–54	18.5	17.9	19.5	21.6	21.3	22.2	22.1	
55–59	17.5	17.5	19.2	21.0	23.0	23.4	21.8	
60–64	10.6	9.1	9.5	9.3	10.7	10.8	10.9	
65–69	14.1	9.6	8.2	7.7	8.3	7.8	8.3	
Smoking, %								<0.001
Never-smoker	86.5	90.2	92.1	93.1	92.5	91.5	88.7	
Ex-smoker	4.7	3.2	3.0	2.9	3.4	4.0	4.5	
<20 cigarettes/day	5.9	4.4	3.4	2.4	2.6	2.6	3.8	
≥20 cigarettes/day	3.0	2.2	1.5	1.6	1.5	1.9	3.1	
Alcohol consumption, %								<0.001
Never-drinker	87.9	85.8	87.2	88.7	89.9	91.6	92.0	
Former-drinker	0.6	0.6	0.7	0.6	0.5	0.7	0.9	
<150 g/week	7.1	9.2	8.4	7.5	6.4	5.2	4.3	
150–<300 g/week	2.9	3.1	2.6	2.2	2.5	1.5	1.4	
≥300 g/week	1.5	1.3	1.1	1.0	0.8	1.1	1.3	
Leisure-time physical activity, %								<0.001
None	54.9	57.1	54.8	55.8	65.1	59.1	62.4	
1–3 times/month	14.3	13.4	14.3	14.8	11.0	12.5	12.0	
1–2 times/week	13.7	13.4	14.4	13.2	10.8	12.4	10.7	
3–4 times/week	9.2	8.0	8.4	8.6	7.0	7.9	7.2	
Almost everyday	7.8	8.2	8.1	7.6	6.2	8.1	7.7	
History of hypertension, %	20.7	23.2	28.4	36.1	44.2	51.7	58.6	<0.001
History of dyslipidemia, %	9.3	12.0	14.5	17.9	20.1	22.8	21.6	<0.001
History of diabetes mellitus, %	3.9	3.6	4.4	5.2	6.9	9.6	13.8	<0.001

^a^Analysis of variance for continuous variables, and Chi-square test for categorical variables.

During a median of 20.0 years of follow-up, 809 and 481 lacunar, 395 and 218 large-artery occlusive, and 568 and 298 cardioembolic strokes were documented in men and women, respectively. Even in the fully-adjusted model that included known mediators, categories of cumulative average BMI were linearly positively associated with all the ischemic stroke subtypes in both sexes (all trend *P* < 0.05), except for cardioembolic stroke in women (trend *P* = 0.14) (Table 2). Relative to men with BMI of 23–<25 kg/m^2^, SHRs of lacunar and large-artery occlusive stroke were significantly lower in those with cumulative average BMI of <19 kg/m^2^ (SHR 0.62; 95% CI, 0.40–0.96; and SHR 0.32; 95% CI, 0.15–0.70, respectively). We observed the significantly increased SHR of cardioembolic stroke in men whose cumulative average BMI was ≥30 kg/m^2^ (SHR 2.14; 95% CI, 1.34–3.41), and that of lacunar stroke in women whose cumulative average BMI were 27–<30 and ≥30 kg/m^2^ (SHR 1.40; 95% CI, 1.05–1.87 and SHR 1.77; 95% CI, 1.19–2.63, respectively). Similarly, women with cumulative average BMI of ≥30 kg/m^2^ had increased SHRs of large-artery occlusive and cardioembolic strokes (SHR 1.90; 95% CI, 1.07–3.37 and SHR 1.89; 95% CI, 1.15–3.11, respectively).

**Table 2.** Sub-distribution hazard ratios and 95% confidence intervals of incident ischemic stroke subtypes according to categories of cumulative average body mass index

		Body mass index (kg/m^2^)							Trend *P*^c^

		<19	19–<21	21–<23	23–<25	25–<27	27–<30	≥30	
Men	person-years	26,828	98,343	194,892	216,690	132,878	66,352	13,798	
Lacunar stroke									
	Number of incidents	23	126	196	219	145	78	22	
	Incidence rate	0.86	1.28	1.01	1.01	1.09	1.18	1.59	
	Multivariable SHR1 (95% CI)^b^	0.63 (0.41–0.98)	1.10 (0.89–1.38)	0.92 (0.76–1.12)	1	1.14 (0.92–1.40)	1.24 (0.95–1.60)	1.70 (1.09–2.64)	0.003
	Multivariable SHR2 (95% CI)^b^	0.58 (0.38–0.90)	1.03 (0.83–1.29)	0.89 (0.74–1.08)	1	1.16 (0.94–1.44)	1.26 (0.97–1.63)	1.68 (1.08–2.62)	<0.001
	Multivariable SHR3 (95% CI)^b^	0.62 (0.40–0.96)	1.09 (0.87–1.36)	0.93 (0.77–1.13)	1	1.14 (0.93–1.41)	1.18 (0.91–1.54)	1.51 (0.96–2.35)	0.007
Large-artery occlusive stroke									
	Number of incidents	7	50	98	127	59	44	10	
	Incidence rate	0.26	0.51	0.50	0.59	0.44	0.66	0.72	
	Multivariable SHR1 (95% CI)^b^	0.32 (0.15–0.69)	0.75 (0.54–1.04)	0.79 (0.61–1.03)	1	0.80 (0.59–1.09)	1.22 (0.86–1.71)	1.37 (0.71–2.63)	<0.001
	Multivariable SHR2 (95% CI)^b^	0.30 (0.14–0.63)	0.69 (0.50–0.97)	0.76 (0.58–0.99)	1	0.83 (0.61–1.13)	1.23 (0.88–1.74)	1.34 (0.70–2.56)	<0.001
	Multivariable SHR3 (95% CI)^b^	0.32 (0.15–0.70)	0.74 (0.53–1.04)	0.80 (0.61–1.05)	1	0.80 (0.59–1.09)	1.15 (0.82–1.61)	1.18 (0.61–2.27)	0.002
Cardioembolic stroke									
	Number of incidents	18	63	145	155	113	54	20	
	Incidence rate	0.67	0.64	0.74	0.72	0.85	0.81	1.45	
	Multivariable SHR1 (95% CI)^b^	0.62 (0.38–1.02)	0.73 (0.54–0.98)	0.93 (0.74–1.17)	1	1.29 (1.01–1.64)	1.27 (0.94–1.73)	2.39 (1.50–3.80)	<0.001
	Multivariable SHR2 (95% CI)^b^	0.60 (0.37–0.99)	0.71 (0.52–0.95)	0.92 (0.73–1.15)	1	1.30 (1.02–1.66)	1.29 (0.95–1.76)	2.36 (1.48–3.77)	<0.001
	Multivariable SHR3 (95% CI)^b^	0.66 (0.40–1.08)	0.75 (0.56–1.01)	0.96 (0.76–1.20)	1	1.27 (1.00–1.62)	1.21 (0.89–1.65)	2.14 (1.34–3.41)	<0.001

Women	person-years	40,758	127,795	228,983	226,509	145,671	86,667	27,263	
Lacunar stroke									
	Number of incidents	16	52	92	127	83	78	33	
	Incidence rate	0.39	0.41	0.40	0.56	0.57	0.90	1.21	
	Multivariable SHR1 (95% CI)^b^	0.57 (0.34–0.96)	0.71 (0.52–0.98)	0.72 (0.55–0.94)	1	0.97 (0.74–1.28)	1.55 (1.17–2.05)	2.14 (1.45–3.16)	<0.001
	Multivariable SHR2 (95% CI)^b^	0.55 (0.32–0.92)	0.71 (0.51–0.98)	0.72 (0.55–0.94)	1	0.97 (0.74–1.28)	1.53 (1.15–2.03)	2.08 (1.41–3.07)	<0.001
	Multivariable SHR3 (95% CI)^b^	0.60 (0.35–1.01)	0.75 (0.55–1.04)	0.74 (0.57–0.97)	1	0.94 (0.71–1.24)	1.40 (1.05–1.87)	1.77 (1.19–2.63)	<0.001
Large-artery occlusive stroke									
	Number of incidents	9	21	46	54	40	32	16	
	Incidence rate	0.22	0.16	0.20	0.24	0.27	0.37	0.59	
	Multivariable SHR1 (95% CI)^b^	0.79 (0.39–1.62)	0.70 (0.42–1.16)	0.85 (0.58–1.27)	1	1.06 (0.70–1.59)	1.40 (0.90–2.17)	2.21 (1.26–3.88)	<0.001
	Multivariable SHR2 (95% CI)^b^	0.74 (0.36–1.52)	0.68 (0.41–1.13)	0.85 (0.57–1.26)	1	1.06 (0.70–1.59)	1.37 (0.89–2.13)	2.12 (1.20–3.72)	<0.001
	Multivariable SHR3 (95% CI)^b^	0.81 (0.40–1.65)	0.73 (0.44–1.21)	0.88 (0.59–1.30)	1	1.02 (0.68–1.54)	1.29 (0.83–2.01)	1.90 (1.07–3.37)	0.003
Cardioembolic stroke									
	Number of incidents	17	39	65	70	41	45	21	
	Incidence rate	0.42	0.31	0.28	0.31	0.28	0.52	0.77	
	Multivariable SHR1 (95% CI)^b^	1.10 (0.65–1.87)	0.97 (0.65–1.43)	0.92 (0.66–1.29)	1	0.85 (0.58–1.25)	1.58 (1.09–2.30)	2.31 (1.41–3.79)	0.006
	Multivariable SHR2 (95% CI)^b^	1.09 (0.64–1.85)	0.97 (0.66–1.44)	0.92 (0.66–1.29)	1	0.85 (0.58–1.25)	1.57 (1.08–2.28)	2.27 (1.38–3.72)	0.007
	Multivariable SHR3 (95% CI)^b^	1.24 (0.73–2.12)	1.07 (0.72–1.59)	0.97 (0.69–1.37)	1	0.81 (0.55–1.18)	1.42 (0.97–2.06)	1.89 (1.15–3.11)	0.14

CI, confidence interval; SHR, sub-distribution hazard ratio.

^a^Crude incidence rate were expressed as rate per 1,000 person-years.

^b^Multivariable SHR1: adjusted for baseline age. Multivariable SHR2: adjusted further for updated smoking, alcohol consumption and leisure-time physical activity. Multivariable SHR3: adjusted further for updated histories of hypertension, dyslipidemia and diabetes mellitus.

^c^Median values of cumulative average body mass index in each categories were used for test of a linear trend across categories.

The stratified analysis by hypertension indicated similar associations of cumulative average BMI with risk of lacunar and cardioembolic stroke regardless of hypertension status in both men and women (*P* for interaction > 0.10 in both men and women) (Table 3). However, the positive association of BMI with risk of large-artery occlusive stroke was not observed in men with hypertension and in women without hypertension (*P* for interaction = 0.01 in men, and 0.08 in women).

**Table 3.** Sub-distribution hazard ratios and 95% confidence intervals for incident ischemic stroke subtypes according to categories of cumulative average body mass index in participants with or without updated history of hypertension

		Body mass index, kg/m^2^							Trend *P*^c^	Interaction *P*^d^

		<19	19–<21	21–<23	23–<25	25–<27	27–<30	≥30		
Men without hypertension										
	Person-years	21,192	74,410	139,031	141,985	80,143	36,261	6,900		
LS	Number of incidents/incidence rate^a^	18/0.85	78/1.05	107/0.77	113/0.80	62/0.77	36/0.99	9/1.30		
	Multivariable SHR (95% CI)^b^	0.73 (0.44–1.21)	1.11 (0.83–1.49)	0.87 (0.67–1.14)	1	1.04 (0.76–1.42)	1.34 (0.91–1.96)	1.63 (0.83–3.23)	0.07	0.90
LAOS	Number of incidents/incidence rate^a^	2/0.09	23/0.31	45/0.32	74/0.52	25/0.31	18/0.50	4/0.58		
	Multivariable SHR (95% CI)^b^	0.12 (0.03–0.47)	0.48 (0.30–0.77)	0.54 (0.37–0.79)	1	0.65 (0.42–1.02)	1.04 (0.63–1.73)	1.18 (0.43–3.23)	<0.001	0.01
CES	Number of incidents/incidence rate^a^	13/0.61	32/0.43	80/0.58	74/0.52	44/0.55	18/0.50	3/0.43		
	Multivariable SHR (95% CI)^b^	0.75 (0.41–1.38)	0.68 (0.44–1.03)	0.98 (0.71–1.35)	1	1.16 (0.80–1.68)	1.10 (0.66–1.84)	0.96 (0.30–3.07)	0.03	0.11
Men with hypertension										
	Person-years	5,636	23,932	55,862	74,705	52,735	30,091	6,898		
LS	Number of incidents/incidence rate^a^	5/0.89	48/2.01	89/1.59	106/1.42	83/1.57	42/1.40	13/1.88		
	Multivariable SHR (95% CI)^b^	0.39 (0.16–0.96)	1.08 (0.77–1.53)	1.00 (0.76–1.33)	1	1.21 (0.91–1.62)	1.07 (0.75–1.53)	1.42 (0.79–2.58)	0.06	
LAOS	Number of incidents/incidence rate^a^	5/0.89	27/1.13	53/0.95	53/0.71	34/0.64	26/0.86	6/0.87		
	Multivariable SHR (95% CI)^b^	0.80 (0.32–2.03)	1.22 (0.76–1.97)	1.20 (0.82–1.76)	1	0.99 (0.64–1.53)	1.32 (0.82–2.12)	1.28 (0.54–3.06)	0.81	
CES	Number of incidents/incidence rate^a^	5/0.89	31/1.30	65/1.16	81/1.08	69/1.31	36/1.20	17/2.46		
	Multivariable SHR (95% CI)^b^	0.47 (0.19–1.16)	0.85 (0.56–1.30)	0.93 (0.67–1.29)	1	1.37 (0.99–1.89)	1.29 (0.87–1.91)	2.69 (1.58–4.59)	<0.001	
Women without hypertension										
	Person-years	32,563	98,548	164,684	146,159	81,717	42,360	11,302		
LS	Number of incidents/incidence rate^a^	10/0.31	31/0.31	54/0.33	48/0.33	29/0.35	22/0.52	13/1.15		
	Multivariable SHR (95% CI)^b^	0.65 (0.33–1.31)	0.88 (0.56–1.38)	0.96 (0.65–1.42)	1	1.02 (0.64–1.62)	1.48 (0.89–2.46)	3.35 (1.76–6.36)	<0.001	0.98
LAOS	Number of incidents/incidence rate^a^	7/0.21	13/0.13	30/0.18	16/0.11	16/0.20	6/0.14	4/0.35		
	Multivariable SHR (95% CI)^b^	1.25 (0.49–3.16)	1.03 (0.49–2.15)	1.58 (0.86–2.89)	1	1.65 (0.82–3.31)	1.11 (0.43–2.89)	2.58 (0.87–7.67)	0.43	0.08
CES	Number of incidents/incidence rate^a^	9/0.28	18/0.18	26/0.16	31/0.21	9/0.11	11/0.26	4/0.35		
	Multivariable SHR (95% CI)^b^	0.95 (0.45–1.99)	0.79 (0.44–1.42)	0.73 (0.43–1.22)	1	0.49 (0.23–1.03)	1.12 (0.56–2.24)	1.56 (0.54–4.52)	0.57	0.53
Women with hypertension										
	Person-years	8,194	29,247	64,299	80,351	63,954	44,307	15,961		
LS	Number of incidents/incidence rate^a^	6/0.73	21/0.72	38/0.59	79/0.98	54/0.84	56/1.26	20/1.25		
	Multivariable SHR (95% CI)^b^	0.54 (0.24–1.25)	0.67 (0.41–1.08)	0.58 (0.39–0.85)	1	0.87 (0.62–1.24)	1.30 (0.92–1.84)	1.23 (0.75–2.02)	<0.001	
LAOS	Number of incidents/incidence rate^a^	2/0.24	8/0.27	16/0.25	38/0.47	24/0.38	26/0.59	12/0.75		
	Multivariable SHR (95% CI)^b^	0.42 (0.10–1.73)	0.58 (0.27–1.24)	0.51 (0.29–0.92)	1	0.76 (0.46–1.28)	1.21 (0.73–2.00)	1.49 (0.76–2.91)	0.001	
CES	Number of incidents/incidence rate^a^	8/0.98	21/0.72	39/0.61	39/0.49	32/0.50	34/0.77	17/1.07		
	Multivariable SHR (95% CI)^b^	1.45 (0.67–3.13)	1.32 (0.78–2.24)	1.19 (0.76–1.85)	1	1.02 (0.64–1.63)	1.63 (1.03–2.59)	2.17 (1.21–3.88)	0.16	

CES, cardioembolic stroke; CI, confidence interval; LAOS, large-artery occlusive stroke; LS indicates lacunar stroke; SHR, sub-distribution hazard ratio.

^a^Crude incident rates were expressed as rate per 1,000 person-years.

^b^Adjusted for baseline age, updated smoking, alcohol consumption, leisure-time physical activity, and histories of dyslipidemia and diabetes mellitus.

^c^Median values of cumulative average body mass index in each categories were used for test of a linear trend across categories.

^d^Interaction was tested by multiplying hypertension and categories of cumulative average body mass index.

The analyses that used baseline BMI yielded generally similar but slightly attenuated results, except for its association with cardioembolic stroke in women (eTable 1). The SHRs of baseline BMI ≥30 kg/m^2^ as well as of lower BMI categories for cardioembolic stroke in women were significant (BMI ≥30 kg/m^2^, SHR 2.55; 95% CI, 1.56–4.19; BMI 19–<21 kg/m^2^, SHR 1.56; 95% CI, 1.04–2.33, quadratic *P* = 0.001).

The updated BMI analyses yielded attenuated results in men (all trend *P* > 0.05) but not in women where a positive linear association with lacunar (trend *P* < 0.001) and large-artery occlusive strokes (trend *P* = 0.03), and a reverse J-shaped association with cardioembolic stroke (quadratic *P* = 0.002) were found. The associations of BMI ≥30 kg/m^2^ for lacunar and large-artery occlusive strokes (HR 2.32; 95% CI, 1.49–3.61 and HR 2.51; 95% CI, 1.31–4.81, respectively) as well as that of BMI <19.0 kg/m^2^ for cardioembolic stroke (HR 2.10; 95% CI, 1.20–3.68) were more evident compared to the findings obtained by cumulative average or baseline BMI analyses (eTable 2).

The sensitivity analyses that regarded undetermined ischemic stroke as lacunar, large-artery occlusive, or cardioembolic strokes, respectively, did not materially change the results (data not shown in table).

## DISCUSSION

In the present prospective study, cumulative average BMI showed a linear and a positive effect on sub-distribution hazard of ischemic stroke across all the subtypes in both sexes except for cardioembolic stroke in women. Compared to persons with BMI of 23–<25 kg/m^2^, men with BMI ≥30 kg/m^2^ had 2.1-times higher risk of cardioembolic stroke, and women with BMI ≥30 kg/m^2^ had 1.8- to 1.9-times higher risks of lacunar, large-artery occlusive, and cardioembolic strokes. Women with BMI of 27–<30 kg/m^2^ had also 1.4-times higher risk of lacunar stroke. These associations were independent of known confounders or mediators, including age, smoking, alcohol consumption, leisure-time physical activity, and histories of hypertension, dyslipidemia, and diabetes mellitus. Linear associations of cumulative average BMI with all ischemic stroke subtypes in men would indicate the existence of no specific cut-points, which implies the importance of preventing a rise in weight in the population for the prevention of ischemic stroke.

We have previously reported a linear positive relation of BMI with the incidence of ischemic stroke only in women in the JPHC Study, with a median follow-up of 7.9 years.^20^ In the present study of extended follow-up with a median of 20.0 years, which focused on ischemic stroke subtypes, we found significant relations of cumulative average BMI with all ischemic stroke subtypes in men. This may be due to increased statistical power, with more cases included in the present study (*n* = 1,181 ischemic stroke cases for the previous analysis and *n* = 1,772 for the present analysis).

Few studies have addressed the association between the degree of obesity and risks of ischemic stroke subtypes.^5^^,^^6^ Our results derived from a relatively lean population (mean baseline BMI: 23.5 kg/m^2^) is primarily consistent with that from a previous study of an American population (mean BMI: 27.8 kg/m^2^), which found BMI quintiles were linearly positively associated with risks of lacunar, non-lacunar, and cardioembolic strokes.^5^ However, the associations in that study were explained by possible mediators, such as measured systolic blood pressure. Another prospective study of Japanese that updated BMI up to five times with those obtained during follow-up examinations reported that the only significant finding was for lacunar stroke incidence in women.^6^ That finding might be consistent with the present analysis that simply updated BMI: the significant associations related to BMI ≥30 kg/m^2^ were found only in women for lacunar and large-artery occlusive strokes. Direct comparison with the ARIC Study regarding possible mediation would be hard since mediators, such as measured systolic blood pressure, total cholesterol, or glucose intolerance, were used in the former, while the majority of the present participants only had self-reported histories of hypertension, dyslipidemia, and diabetes mellitus. Nevertheless, we should consider that residual confounding (mediation) would be likely in the analyses where only self-reported histories were adjusted. However, if the association existed between BMI and ischemic stroke subtypes, independent of known mediators, possible explanations would include insulin resistance, endothelial dysfunction, or a low-grade chronic inflammation state.^21^^,^^22^ In any case, the present study suggests the importance of weight control for the prevention of all ischemic stroke subtypes.

The excess risks of lacunar and large-artery occlusive strokes for BMI ≥30 kg/m^2^ category compared to the reference category (23–<25 kg/m^2^) seemed more evident in women than in men. This might be due to some unmeasured stroke risk factors that are reportedly more strongly related to obesity in women than in men, such as low-grade systematic inflammation^23^^,^^24^ and prothrombotic factors.^25^ In any case, the present results imply that avoidance of obesity offers the potential of reducing lacunar and large-artery occlusive strokes in women.

The updated BMI analysis showed that the risk of cardioembolic stroke for the lowest BMI category with cardioembolic stroke was significantly elevated in women. This finding might correspond to a U-shaped association reported between BMI and atrial fibrillation,^26^ the most important risk factor for cardioembolic stroke.^27^^,^^28^ Furthermore, low body weight was reportedly a significant risk factor for stroke incidence in male and female Japanese patients with atrial fibrillation.^29^ Further studies that obtained atrial fibrillation as well as use of antiplatelet medication, which may modify the association of atrial fibrillation with risk of cardioembolic stroke^30^ are warranted.

The stratified analyses by hypertension yielded somewhat puzzling results: the lack of association between cumulative average BMI and risk of large-artery occlusive stroke in men with hypertension and in women without hypertension. The lack of the association in men with hypertension may be due to the higher probability of treatment for dyslipidemia and diabetes in the higher categories of BMI by local physicians.^31^^,^^32^ This tendency might have contributed to the prevention of large-artery occlusive stroke because dyslipidemia and diabetes, as well as hypertension, are major risk factors for this stroke subtype.^33^^,^^34^ Another reason for the lack of that association might be related to the fact that hypertension is the single strongest risk factor for large-artery occlusive infarction, the impact of BMI could be masked and not be properly evaluated in men with hypertension. In women without hypertension, the lack of the association between cumulative average BMI and risk of large-artery occlusive stroke might merely be by chance, probably because the number of large-artery occlusive stroke cases was small.

Simply speaking, the analyses that only used baseline or updated BMI produced attenuated results, which implies that cumulative average BMI captured important and relevant aspect of long-term effect of BMI on the sub-distribution hazard of ischemic stroke subtypes better than single-point measures. However, this was not true for women. The increased risk of cardioembolic stroke observed for women with BMI ≥30 kg/m^2^ as well as for those with 19–<21 kg/m^2^ were more apparent in the baseline BMI analysis compared to the cumulative average BMI analysis. In addition, the associations of BMI ≥30 kg/m^2^ with risks of lacunar and large-artery occlusive strokes, as well as the association of BMI <19 kg/m^2^ with risk of cardioembolic stroke, were more evident in the updated BMI analysis compared to the cumulative average BMI analysis. Women might be more susceptible to a short-term effect of BMI, which is indicated by the fact that women gain weight with aging on average.^35^

The strengths of the present study include valid identification of sufficient numbers of incident ischemic stroke subtypes that enabled us to conduct sex-specific analyses or stratified analyses by hypertension. Moreover, we examined three BMI variables in relation to ischemic stroke subtype risks, somewhat different results of which would aid better comparisons among studies. Also, we took competing risk of death into account to obtain more accurate estimates of the associations.

The present study has potential limitations. First, BMI was calculated from self-reported body weight and height. Although the validity of self-reported height and body weight has been reported,^36^^,^^37^ overweight or obese subjects underestimated their body weight and underweight subjects overestimated their body weight,^38^ which would be related to attenuation of the observed associations. Second, the information about atrial fibrillation was not available. Although inconsistencies exist in the literature,^39^^–^^41^ low as well as high BMI were reportedly associated with high risk of atrial fibrillation.^41^^,^^42^ The present reverse J-shaped association of BMI with cardioembolic stroke risk might have been partly explained via atrial fibrillation. However, the adjustment for self-reported arrhythmia or the exclusion of those with self-reported arrhythmia did not materially change the reverse J-shaped association. Third, we were unable to evaluate to what extent the observed association was mediated through measured blood pressure or blood levels of glucose and lipids, since less than 25% subjects had complete data. Previous studies in Western populations have reported that these mediators alone or together entirely explained the effect of BMI on incident lacunar, nonlacunar, and cardioembolic stroke.^5^ Further studies in East Asian population are needed to clarify whether the significant association found in the present study would be explained via those measured potential mediators.

In conclusion, cumulative average BMI showed a positive linear effect on sub-distribution hazards of lacunar, large-artery occlusive, and cardioembolic strokes in both sexes, except for cardioembolic stroke in women. There were approximately two-fold excess risk of cardioembolic stroke in both sexes and of lacunar and large-artery occlusive strokes in women for BMI ≥30 kg/m^2^ compared to BMI 23–<25 kg/m^2^.

## APPENDIX A. SUPPLEMENTARY DATA

eTable 1. Sub-distribution hazard ratios and 95% confidence intervals of incident ischemic stroke subtypes according to categories of baseline body mass index

eTable 2. Hazard ratios and 95% confidence intervals of incident ischemic stroke subtypes according to categories of updated body mass index
