# Supplementary material for: Body Mass Index and Risks of Incident Ischemic Stroke Subtypes: The Japan Public Health Center-Based Prospective (JPHC) Study
Source: J Epidemiol. 2019 Sep 5;29(9):325–33. doi: 10.2188/jea.JE20170298 (PMC6680058; doi:10.2188/jea.JE20170298)
Supplement: Supplementary file 1 [file je-29-325-s001.pdf]

eTable 1. Sub-distribution hazard ratios and 95% confidence intervals of incident ischemic stroke subtypes according to categories of baseline body mass index

		Body mass index, kg/m^2							Trend P[c]
		<19	19- <21	21- <23	23- <25	25- <27	27- <30	≥30	
Men, person-years		27,803	106,145	192,782	209,841	1295,98	67,225	16,387	
LS	Number of incidents	20	131	193	213	144	82	26	
	Incidence rate[a]	0.72	1.23	1.00	1.02	1.11	1.22	1.59	
	Multivariable SHR (95% CI)[b]	0.56 (0.35–0.89)	1.10 (0.88–1.37)	0.93 (0.77–1.13)	1	1.11 (0.90–1.37)	1.20 (0.93–1.55)	1.50 (1.00–2.27)	0.005
LAOS	Number of incidents	11	51	103	109	55	55	11	
	Incidence rate[a]	0.40	0.48	0.53	0.52	0.42	0.82	0.67	
	Multivariable SHR (95% CI)[b]	0.60 (0.32–1.12)	0.82 (0.59–1.15)	0.97 (0.74–1.27)	1	0.84 (0.61–1.16)	1.61 (1.17–2.23)	1.25 (0.67–2.36)	0.004
CES	Number of incidents	21	62	157	142	97	71	18	
	Incidence rate[a]	0.76	0.58	0.81	0.68	0.75	1.06	1.10	
	Multivariable SHR (95% CI)[b]	0.82 (0.52–1.31)	0.75 (0.55–1.01)	1.10 (0.88–1.38)	1	1.14 (0.88–1.48)	1.64 (1.23–2.19)	1.68 (1.03–2.76)	<0.001
Women, person-years		44,615	133,843	230,605	219,265	137,760	88,580	28,979	
LS	Number of incidents	21	56	92	128	76	71	37	
	Incidence rate[a]	0.47	0.42	0.40	0.58	0.55	0.80	1.28	
	Multivariable SHR (95% CI)[b]	0.78 (0.49–1.24)	0.79 (0.58–1.09)	0.72 (0.55–0.93)	1	0.86 (0.65–1.15)	1.17 (0.87–1.57)	1.82 (1.25–2.66)	<0.001
LAOS	Number of incidents	8	23	50	46	43	33	15	

	Incidence rate[a]	0.18	0.17	0.22	0.21	0.31	0.37	0.52	
	Multivariable SHR (95% CI)[b]	0.81 (0.38–1.74)	0.92 (0.56–1.52)	1.11 (0.74–1.65)	1	1.33 (0.87–2.02)	1.42 (0.90–2.23)	1.87 (1.04–3.38)	0.01
CES	Number of incidents	15	43	66	53	50	47	24	
	Incidence rate[a]	0.34	0.32	0.29	0.24	0.36	0.53	0.83	
	Multivariable SHR (95% CI)[b]	1.43 (0.81–2.53)	1.56 (1.04–2.33)	1.27 (0.88–1.82)	1	1.32 (0.90–1.95)	1.77 (1.19–2.62)	2.55 (1.56–4.19)	0.06

CES, cardioembolic stroke; CI, confidence interval; LAOS, large-artery occlusive stroke LS, lacunar stroke; SHR, sub-distribution hazard ratio.

[a] Crude incidence rates were expressed as rate per 1,000 person-years.

[b] Adjusted for baseline age, smoking, alcohol consumption, leisure-time physical activity, and histories of hypertension, dyslipidemia and diabetes mellitus.

[c] Median values of baseline body mass index in each categories were used for test of a linear trend across categories.

eTable 2. Hazard ratios and 95% confidence intervals of incident ischemic stroke subtypes according to categories of updated body mass index

		Body mass index, kg/m^2							Trend P[c]
		<19	19- <21	21- <23	23- <25	25- <27	27- <30	≥30	
Men, person-years		23,967	78,662	138,760	155,903	97,338	53,351	12,890	
LS	Number of incidents	20	89	130	149	88	60	15	
	Incidence rate[a]	0.83	1.13	0.94	0.96	0.90	1.12	1.16	
	Multivariable HR (95% CI)[b]	0.67 (0.42–1.07)	1.04 (0.79–1.35)	0.92 (0.73–1.16)	1	1.01 (0.78–1.32)	1.22 (0.90–1.65)	1.29 (0.76–2.21)	0.17
LAOS	Number of incidents	9	34	61	79	46	20	7	
	Incidence rate[a]	0.38	0.43	0.44	0.51	0.47	0.37	0.54	
	Multivariable HR (95% CI)[b]	0.58 (0.29–1.16)	0.77 (0.51–1.15)	0.82 (0.59–1.15)	1	0.97 (0.67–1.39)	0.72 (0.44–1.18)	1.09 (0.50–2.36)	0.07
CES	Number of incidents	20	46	83	113	54	42	10	
	Incidence rate[a]	0.83	0.58	0.60	0.72	0.55	0.79	0.78	
	Multivariable HR (95% CI)[b]	0.87 (0.54–1.40)	0.72 (0.51–1.01)	0.77 (0.58–1.03)	1	0.82 (0.59–1.13)	1.14 (0.80–1.63)	1.19 (0.62–2.28)	0.13
Women, person-years		35,564	94,942	164,119	157,561	103,767	67,176	23,152	
LS	Number of incidents	11	37	59	65	64	57	29	
	Incidence rate[a]	0.31	0.39	0.36	0.41	0.62	0.85	1.25	
	Multivariable HR (95% CI)[b]	0.68 (0.36–1.29)	0.99 (0.66–1.48)	0.91 (0.64–1.30)	1	1.35 (0.95–1.91)	1.73 (1.21–2.47)	2.32 (1.49–3.61)	<0.001
LAOS	Number of	6	11	22	28	23	15	14	

	incidents								
	Incidence rate[a]	0.17	0.12	0.13	0.18	0.22	0.22	0.60	
	Multivariable HR (95% CI)[b]	0.89 (0.36–2.16)	0.70 (0.35–1.42)	0.81 (0.46–1.41)	1	1.11 (0.64–1.92)	1.02 (0.54–1.91)	2.51 (1.31–4.81)	0.03
CES	Number of incidents	19	21	37	37	18	26	11	
	Incidence rate[a]	0.53	0.22	0.23	0.23	0.17	0.39	0.48	
	Multivariable HR (95% CI)[b]	2.10 (1.20–3.68)	1.01 (0.59–1.73)	1.02 (0.65–1.62)	1	0.64 (0.37–1.13)	1.32 (0.80–2.19)	1.44 (0.73–2.83)	0.86

CES, cardioembolic stroke; CI, confidence interval; HR, hazard ratio; LAOS, large-artery occlusive stroke; LS, indicates lacunar stroke.

[a] Time-dependent crude incidence rates were expressed as rate per 1,000 person-years within each 5-year follow-up interval.

[b] Adjusted for baseline age, updated smoking, alcohol consumption, leisure-time physical activity, and histories of hypertension, dyslipidemia and diabetes mellitus.

[c] Median values of updated body mass index in each categories were used for test of a linear trend across categories.
